# Continuous Synthesis of Nanoscale Emulsions by Vapor Condensation (EVC)

**DOI:** 10.1002/advs.202307443

**Published:** 2024-02-14

**Authors:** Sushant Anand, Vincent Galavan, Mahesh Uttamrao Mulik

**Affiliations:** ^1^ Department of Mechanical and Industrial Engineering University of Illinois at Chicago 842 West Taylor St. Chicago IL 60607 USA; ^2^ Department of Nuclear Science & Engineering Massachusetts Institute of Technology 77 Massachusetts Ave Cambridge MA 02139 USA; ^3^ Spruce Up Industries Undri – Pisoli Rd Pune Maharashtra 411060 India

**Keywords:** condensation, continuous synthesis, emulsions

## Abstract

Emulsions are widely used in many industrial applications, and the development of efficient techniques for synthesizing them is a subject of ongoing research. Vapor condensation is a promising method for energy‐efficient, high‐throughput production of monodisperse nanoscale emulsions. However, previous studies using this technique are limited to producing small volumes of water‐in‐oil dispersions. In this work, a new method for the continuous synthesis of nanoscale emulsions (water‐in‐oil and oil‐in‐water) is presented by condensing vapor on free‐flowing surfactant solutions. The viability of oil vaporization and condensation is demonstrated under mild heating/cooling using diverse esters, terpenes, aromatic hydrocarbons, and alkanes. By systematically investigating water vapor and oil vapor condensation dynamics on bulk liquid‐surfactant solutions, a rich diversity of outcomes, including floating films, nanoscale drops, and hexagonally packed microdrops is uncovered. It is demonstrated that surfactant concentration impacts oil spreading, self‐emulsification, and such behavior can aid in the emulsification of condensed oil drops. This work represents a critical step toward advancing the vapor condensation method's applications for emulsions and colloidal systems, with broad implications for various fields and the development of new emulsion‐based products and industrial processes.

## Graphical abstract

A new method for continuous synthesis of nanoscale emulsions is presented using vapor condensation on surfactant solutions. Investigations of water and oil vapor condensation reveal various outcomes, including floating films, nanoscale drops, and hexagonally packed microdrops ‐ dependent upon the surfactant concentration. The method offers potential applications in emulsions and colloidal systems, impacting various fields, and industrial processes.

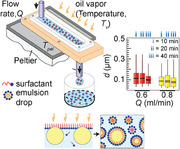

## 1 Introduction

The incredible versatility of emulsions^[^ ^1^ ^]^ across a broad spectrum of industries,^[^ ^2^ ^]^ including drug delivery,^[^ ^3^ ^]^ cosmetics,^[^ ^4^ ^]^ foods,^[^ ^5^ ^]^ fuels,^[^ ^6^ ^]^ oil recovery,^[^ ^7^ ^]^ and materials synthesis^[^ ^8^ ^]^ has been a source of inspiration to study them for many decades.^[^ ^9^ ^]^ Considerable efforts have been made to develop techniques to formulate emulsions with nanometric drop sizes as this improves emulsion stability, rheology, and applicability.^[^ ^2^, ^10^ ^]^ In this regard, recently a new method was introduced to synthesize nanoscale‐sized emulsions in a single step by condensing water vapor on mixtures of oil and surfactants^[^ ^11^ ^]^ as well as oil and particles.^[^ ^12^ ^]^ By extending this technique, nanoparticles have also been successfully synthesized.^[^ ^13^ ^]^ The ease of making water in oil (*w/o*) emulsions through condensation stems from different interfacial phenomena acting in confluence to submerge drops nucleated at the oil–air interface.^[^ ^11^, ^12^, ^14^ ^]^ Water drops condense on the oil–air interface via heterogeneous nucleation and thereafter grow via vapor diffusion and coalescence.^[^ ^14^, ^15^ ^]^ This growth depends on the nucleation rate, and the spreading behavior of oil on water (in the presence of air), which is thermodynamically represented by the spreading coefficient *S_ow_*_(_ *_a_*_)_  =  *γ_wa_* – *γ_oa_* – *γ_ow_* (*γ_wa_*, *γ_ow,_* and *γ_oa_* refer to the water/air, water/oil, and oil/air interfacial tensions, respectively). For oil mixtures with *S_ow_*_(_ *_a_*_)_>0, an oil film rapidly spreads on nucleated drops, causing them to submerge within the oil.^[^ ^14^ ^]^ If a sufficient concentration of surfactants/nanoparticles is present in the oil, they can rapidly absorb on the submerged water droplets, impeding their coalescence, and leading naturally to the formation of stable emulsions.^[^ ^11^, ^12^ ^]^ Emulsions by vapor condensation (EVC) technique has several advantages, for instance, it has a large throughput, and is potentially scalable^[^ ^12^ ^]^, similar to many high‐energy techniques like sonication.^[^ ^16^ ^]^ Additionally, it is highly energy‐efficient^[^ ^12^ ^]^ when compared to existing homogenization‐based methods, similar to many low‐energy techniques such as phase inversion^[^ ^17^ ^]^, microfluidics^[^ ^18^ ^]^, and Ouzo effect^[^ ^19^ ^]^, or jetting/impacting drops.^[^ ^20^ ^]^ Sonicators and high‐pressure homogenizers generate high local temperature and pressure gradients that can cause depolymerization, denaturation, and aggregation of biomolecules, negatively impacting their function.^[^ ^21^ ^]^ In contrast, droplet formation in the EVC technique occurs at low temperatures by default and thus precludes such harmful effects. Microemulsion techniques yield nanoscale emulsions but have limited scalability for industrial production and a narrow compositional range necessitating specific emulsifiers to achieve ultra‐low oil/water surface tension, some of which may be toxic, restricting their application in pharmaceutical, and food‐related contexts. In contrast, the EVC technique does not require ultra‐low surface tensions allowing for greater flexibility in creating nanoscale drops.

The early‐stage work demonstrated the potential benefits of the EVC technique, but addressing various obstacles is essential to promote its widespread adoption in the industry. For instance, vaporizing and condensing water is easy, but it is unclear what types of oils can be vaporized easily and whether simple oil in water (*o/w*) or complex emulsions (oil/water/oil or water/oil/water) can be formulated by vapor condensation. The formation of *w/o* emulsions relies on the fact that while some oils spread on water (i.e., *S_ow_*_(_ *_a_*_)_>0), water does not spread on the oils (i.e., spreading coefficient of water on oil in the presence of air, *S_wo_*_(_ *_a_*_)_<0) due to its large surface tension. This phenomenon is also an impediment in synthesizing *o/w* emulsions because water does not have a natural tendency to cloak and submerge the oil condensate. Besides, vapor condensation on surfactant solutions of oil and water has a fundamental difference. Oil‐soluble surfactants do not preferentially partition at the oil–air interface, so water drops nucleate on the pure oil–air interface, whereas, water‐soluble surfactants do partition at the water‐air interface, so oil condenses on the surfactant‐covered water surface. Consequently, oil‐on‐water condensation may depend upon liquid‐on‐liquid spreading dynamics to a greater degree than water‐on‐oil condensation. Finally, additives such as proteins, drug molecules, and polymers are added to emulsified drops based on the specific requirements of their applications. However, many of these additives cannot be easily vaporized without losing their useful functionalities. Even if some additives are vaporizable, it is unclear how they can be incorporated effectively in condensed drops, which represent the purest form of the condensed material by their very nature.

This work aims to address the first two impediments discussed above. We propose a new method for the continuous synthesis of emulsions with nanoscale‐sized drops by condensing vapor on free‐flowing surfactant solutions. This design allows for easy scalability to produce emulsions in any volume. We systematically investigate how condensation in conjunction with other interfacial mechanisms, namely the oil spreading dynamics, and self‐emulsification can lead to *o/w* emulsions even in the absence of the cloaking/submergence mechanism that is typical of *w/o* emulsion. Our findings provide important new steps toward guiding the applications of the vapor condensation method for emulsions and colloidal systems.

## 2 Results and Discussion

**Figure 1:**
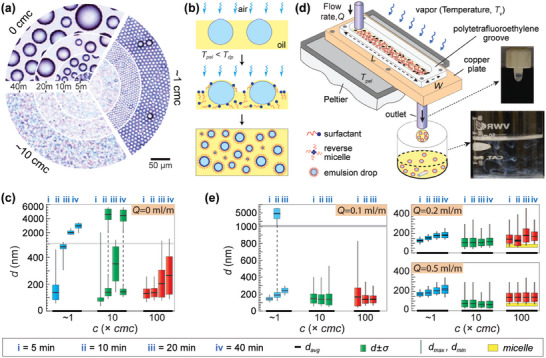
a) Images showing size evolution of condensed water vapor droplets on a stagnant subcooled oil‐surfactant solution (oil phase: Kerosene, surfactant: Span‐80) at different surfactant concentrations. b) Schematic illustrating the underlying mechanism of emulsion formation via condensation. c) Droplet size distributions of *w/o* emulsion on stagnant oil (zero flow rate). d) Schematic of the experimental setup for the continuous emulsification of condensing drops and inset images showing outlet nozzle and the collection beaker in which dripping/ flowing emulsion drops are collected. e) Emulsion size distribution obtained as a function of surfactant concentration, flow rate, and condensation time.^[^ ^15^ ^]^ In all experiments, *T_dp_* ≈17 ± 1 °C, *T_pel_* = 2 ± 0.5 °C.

W/O emulsions by condensation on a stagnant oil‐surfactant solution. We first describe the outcomes of condensation on stagnant pools of pure oil and oil‐surfactant solutions. We selected kerosene as the oil phase due to its natural ability to spread on water and Span‐80 (critical micelle concentration, cmc *=* 0.2 mm) as the oil‐soluble surfactant. The growth behavior of droplets on solutions with different Span‐80 concentrations (0, 1, 10, and 100 ×cmc) was visualized using an optical microscope under identical conditions (Dew point temperature, *T_dp_* ∼17 ± 1 °C, Peltier temperature, and *T_pel_* = 2 ± 0.5 °C, see Experimental Section for more details). **Figure** **1**a presents the image sequence depicting the time evolution (radially outward) of condensed water drops on kerosene solutions with different Span‐80 concentrations (0, 1, and 10 ×cmc), and a zoomed‐out view is presented in Figure S1 (Supporting Information). On pure oil, water droplets rapidly grow through coalescence, and their polydispersity increases over time. For reasons that remain to be fully understood, the drops anchored at the oil–air interface, as evidenced by their remaining within the microscope's focal plane, even though a three‐phase contact line was absent (due to *S_ow(a)_* >0). Upon collecting the solution in a beaker, the oil quickly separates from the condensed droplets, as kerosene is less dense than water. On the ≈1×cmc solution, condensing water droplets self‐arranged to form a beautiful hexagonally close‐packed pattern with droplet size increasing over time and minimal polydispersity variation. The condensation patterns in the 10*×*cmc and 100*×*cmc solutions were hazy, and no visibly distinguishable drops were observed. As depicted in Figure 1b, in these cases, the drops first nucleate at the oil–air interface, and are then instantly covered by the oil‐surfactant solution that then causes them to submerge within. As more drops undergo this process, they undergo a competition between coalescence and surfactant absorption, eventually forming stable emulsions like previous works.^[^ ^11^, ^14^ ^]^ To compare how drop sizes change over time for the micellar oil solutions, we condensed water vapor on them for a fixed period, henceforth referred to as condensation time, *t_con_*. After keeping the 10*×*cmc and 100*×*cmc solutions on the Peltier for the duration of *t_con_* = 5, 10, 20, and 40 min respectively, the resulting emulsified solution was immediately collected and analyzed using dynamic light scattering (DLS). For the ≈1×cmc case, image analysis was used since the drops were of micrometric sizes. The variation of emulsion size with *t_con_* is presented in Figure 1c. The basis for converting the DLS data to the bar–chart graph is shown in Figure S2 (Supporting Information). At 1×cmc, the mean size of the collected emulsion is in the nanoscale range for short condensation time (*t_con_* ≈5 min) but grew to micron sizes at later times, as observed in Figure 1a. At 10×cmc, most of the solution comprises emulsion drops in the nanoscale size range, with a second small peak observed in the micron range. On 100×cmc solutions, nanometric‐sized emulsions partially overlap with reverse micelles (≈56 nm) form, and their average size increases with condensation time. In 10×cmc and 100×cmc solutions, nanosized drops are formed due to the rapid adsorption of surfactants on water droplets condensing at the oil–air interface either through micelle dissociation or direct micelle adsorption, thereby ceasing drop coalescence. However, the surfactant concentration progressively decreases as they absorb onto the continuously nucleating drops, leading to surfactant depletion near the oil–air interface^[^ ^11^ ^]^, and causing the drop size to increase over time.

W/O emulsions by condensation on a flowing oil‐surfactant solution. To overcome the issue of surfactant depletion present in the previous experimental configuration and to continuously synthesize emulsions, we introduce a new technique wherein water droplets condense on an oil‐surfactant solution flowing unidirectionally (at a rate of *Q *ml min^−1^) within a channel placed on a Peltier surface. Briefly, the channel was created by securely attaching a hollowed polytetrafluoroethylene (PTFE) plate (length *L* = 14 cm and width *W* = 3 mm) to an aluminum plate, and the oil was pumped at one of its ends through a needle attached to a syringe pump. A hole connected to a needle at the other end of the aluminum plate served as an outlet for the flowing solution. A schematic of this setup is shown in Figure 1d and setup details/ experiment protocols are provided in the Experimental Section. A representative video of the emulsion collection is shown in Movie S1 (Supporting Information). We hypothesized that the oil flow would facilitate the water condensation on fresh oil surface over time and prevent coalescence between new and older generations of condensed droplets by transporting the latter away from the Peltier‐cooled channel region leading to monodisperse nano‐sized emulsions. To test this hypothesis, we conducted experiments at 1, 10, and 100 ×cmc for identical *t_con_* (5, 10, 20, and 40 min) by varying the flow rate (*Q*) of the injected oil solution at 0.1, 0.2, and 0.5 mL min^−1^. The resulting emulsified solution in each case was collected in a beaker positioned outside the device and immediately analyzed for size using DLS. The results for the three different flow rates and different *t_con_* are shown in Figure 1e, while the raw DLS data is shown in Figure S3 (Supporting Information) along with pictures of collected emulsions. The results demonstrate that for all the surfactant concentrations, the emulsion sizes were smaller and significantly more monodisperse compared to the case of zero flow rate (i.e., condensation on a stagnant pool) and compared to emulsions formulated by steam injection.^[^ ^22^ ^]^ Remarkably, the emulsion sizes remained relatively constant and nearly identical over time for 10×cmc and 100×cmc solutions at all the tested flow rates. Increasing the flow rate produced emulsions with higher solution transparency because of the reduced fraction of condensed droplets in the solution. For the 10×cmc case, the emulsion size increased from Day‐0 to‐7 (likely due to lower surfactant concentration) but stabilized afterward (Figure S4, Supporting Information). Note that we intentionally made a smaller setup herein to investigate the condensation dynamics. However, this design can be easily scaled to generate any desired quantity of emulsions.

### 2.1 Surfactant Adsorption Dynamics

The kinetics of the surfactant adsorption on condensed droplets in micellar solutions are complicated, involving several processes that occur over different time scales, including diffusion of unaggregated (i.e., monomer) surfactant molecules, micelle diffusion, micelle unpacking, and adsorption.^[^ ^23^ ^]^ Barring such complexities, a simpler picture can be obtained in the limit of the surfactant concentration approaching its cmc in a quiescent fluid. Previous studies have shown that surfactant transport on droplets <≈10 µm is kinetically controlled, i.e., the fastest timescale relates to surfactant diffusion (*t_d_*).^[^ ^24^ ^]^ The characteristic length scale for diffusion, called the adsorption depth (*δ_p_*) can be estimated as *δ_p_* = 𝛤_∞_/cmc, where 𝛤_∞_ is the maximum surfactant interfacial coverage.^[^ ^24^, ^25^ ^]^ For Span‐80, 𝛤_∞_ ≈4.42 *× *10^–6^ mol. m^−2^ from where we get *δ_p_* ≈22 µm, much larger than the average drop diameter, *d*∼𝒪O(100 nm) formed via the EVC technique. For drops with *d*/2*δ_p_* ≪1, *t_d_* = *dδ_p_*/2*D*_diff_ where *D*_diff_ is the surfactant monomer diffusion coefficient (≈5 *× *10^–10^ m^2^ s^−1^ for Span‐80 in oil).^[^ ^24^, ^25^ ^]^ Using this relation, we find that *t_d_* ≈80 to 800 µs for 10–100 nm diameter droplets, implying that droplets are promptly covered by surfactants upon formation via condensation.

Another aspect of interest to the EVC technique is the fraction (Φ) of the dispersed phase in the continuous phase. Practically, Φ can be estimated by carefully measuring the weight gain (*m_w_*) of an oil‐surfactant solution after condensing over the period *t_con_*. For a total oil volume given by *V_o_* = *Q*t*_con_*, Φ can then be calculated as Φ = *m_w_*/(*ρ_o_Qt_con_*). Alternatively, Φ can also be estimated by using the nucleation rate, *J* #/m^2^s over the bulk liquid. Drops nucleating on the surface are instantly cloaked, so new drops nucleate on a fresh oil surface while submerged drops engage in a struggle between coalescence, which enhances their growth, and surfactant adsorption, which seeks to restrict it. Assuming a steady‐state rate of nucleation on the surface, the condensed volume is given by *V_w_* = *J*A*_s_* t*_co_*_n_vol*_drop_* where vol*_drop_* is the volume of the critical nuclei, and *A_s_* is the surface area over that condensation occurs (*A_s_* = *LW* for our case). The total volume of oil is *V_o_* = *Qt_con_*, and the molar surfactant consumed during that period is *n_s_* = (*a×*cmc)*×Qt_con_* micromoles where (*a×*cmc) is the amount of surfactant as a factor of its cmc (mm). Using these, we find that Φ = *J∙vol_drop_*/(*Q*/*A_s_*), which implies that increasing the flow rate leads to a smaller emulsion fraction in the solution. Separate calculations (discussed in Supporting information) suggest that the nucleation rate is ≈O (10^17^ #/m^2^ s) for our experimental conditions, and assuming a radius of 5 nm, we find that Φ = 1% for the flow rate of *Q* = 0.1 mL min^−1^. The emulsion fraction can be increased, for example, by reducing the flow rate and increasing the nucleation rate, and theoretically, high‐internal phase emulsions can be achieved also. Such aspects were not explored herein.

### 2.2 Choice of Oils, their Spreading Behavior, and Condensation on Stagnant Pure Water

**Figure 2:**
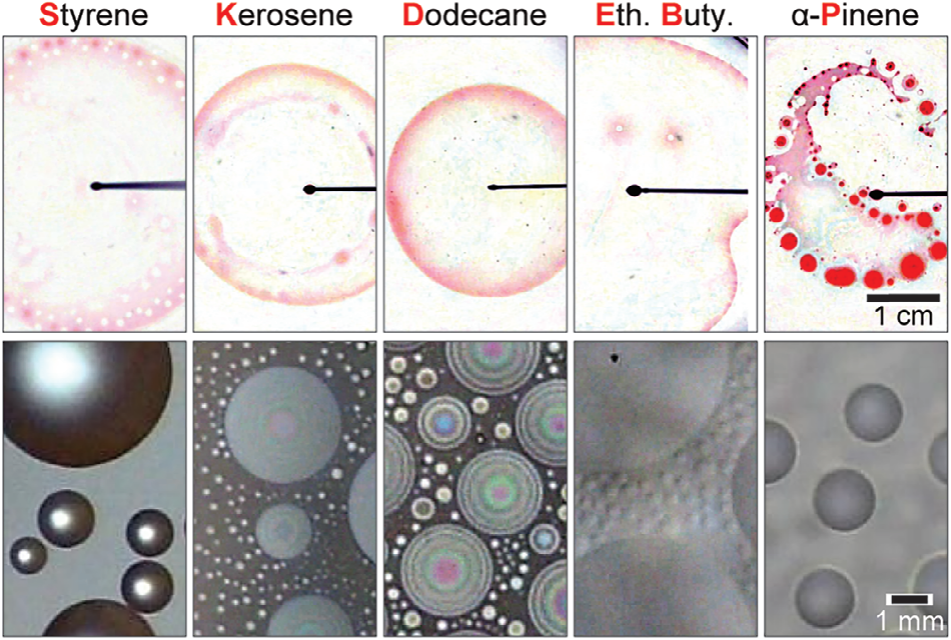
Top row shows a representative image of an oil drop spread on pure water in ambient conditions. Bottom row shows a representative image of oil drops condensed on pure water held at 10 ± 0.5 °C. Each oil was heated to ≈30 °C for vaporization.

As a first step toward synthesizing *o/w* emulsions, we sought to identify suitable oils that would undergo vaporization with mild heat (30–50  °C) and selected five oils with vapor pressures varying from 0.088 to 11 mmHg at 25 °C that met our criteria. These oils are i) dodecane, an alkane commonly used as fuel, solvent, lubricant, and in chemical synthesis; ii) kerosene, an alkane mixture commonly used as fuel, solvent, and in insecticides; iii) styrene, an aromatic hydrocarbon commonly used in the production of plastics, resins, synthetic rubber, and as a precursor to polystyrene; iv) ethyl butyrate, an ester commonly used as a flavoring agent in the food industry, in fragrances, pesticides, and solvents; and v) α‐pinene, a terpene often used in the fragrance and flavor industry and known for its anti‐inflammatory and analgesic properties. Their properties, including their spreading coefficients on the water, are given in Table S1 (Supporting Information) in the Experimental Section. Among these oils, dodecane, and α‐pinene have S_ow(a)_ < 0, so they are expected to form a lens on water. Styrene, kerosene, and ethyl butyrate have *S_ow_*_(_ *_a_*_)_>0, so are expected to form a film or pseudo‐lens. To test these predictions, we visualized the behavior of gently deposited oil drops on a pure water surface. These tests showed that dodecane existed as a lens on water but the α‐pinene drop showed instabilities and fragmented. Kerosene is a combination of mostly C_9_–C_16_ alkanes and the Hamaker constant is positive for liquid alkanes at room temperature,^[^ ^26^ ^]^ so as expected, its droplet spread on water and formed pseudo‐lens.^[^ ^27^ ^]^ The styrene droplet showed a larger spreading radius and instabilities at its rim whereas the ethyl butyrate droplet ruptured after spreading over water. The ethyl butyrate behavior is likely related to its large solubility with water and higher vapor pressure (see Table S1, Supporting Information). Finally, for α‐pinene, the instabilities observed could be related to its high vapor pressure and evaporation. In the next step, we tested the condensation of the chosen oils onto stagnant pure water. Oil vapors can form nanoscopic (≈2 nm) films at the water‐air interface through absorption,^[^ ^28^ ^]^ but emulsion formation necessitates a substantial amount of oil, attainable through condensation. A special copper chamber was fabricated to simultaneously vaporize the oil and visualize its condensation on the water under a microscope (see Figure S5 and Experimental Section, Supporting Information). All the experiments were conducted at 30 °C heater temperature and 10 °C Peltier temperature. The results from top‐down imaging show that all oils condensed on the water surface in either lens or pseudo‐lens states (**Figure** **2**, bottom row; Movie S2, Supporting Information). The formation of oil film/drops was synonymous with the oil spreading experiments. The transparency and lens/pseudo‐lens shape of dodecane and kerosene drops respectively enabled them to reflect and refract light, resulting in notable thin‐film interference patterns. Unlike α‐pinene spreading experiments, the condensed α‐pinene droplets did not rupture. These findings establish that vaporizing and condensing oil on an aqueous surface is a feasible process even at mild heating /cooling conditions.

### 2.3 Oil Drop Dynamics on Triton X‐100 Solutions

**Figure 3:**
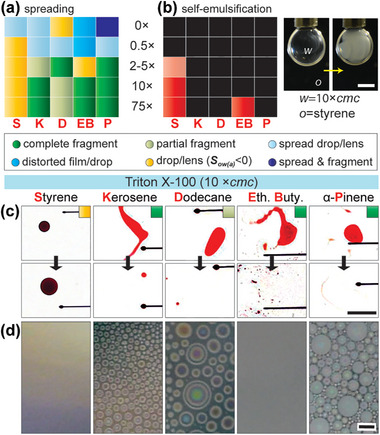
The regime map for outcomes of pure oil drops on water‐Triton X‐100 mixtures related to the a) spreading behavior and b) self‐emulsification in ambient conditions. Also shown in b) is an example of self‐emulsification within a water‐Triton X‐100 drop. c) Images showing the outcome of oil spreading behavior in ambient conditions. d) Representative images of typical condensation behavior of oils on water‐surfactant solution held at 10 ± 0.5 °C. Each oil was heated to ≈30 °C for vaporization. Scale bar in b) 1 mm, c) 1 cm, and all images in d) 100 µm.

Two aspects of oil‐water dynamics of relevance here are the spreading kinetics and the possibility of the spontaneous self‐emulsification that can happen for certain oil‐water‐surfactant combinations. Identifying the absence or occurrence of both mechanisms is vital for understanding their importance (or lack thereof) during the EVC technique since both can also form emulsions. We selected Triton X‐100 (measured cmc = 0.24 mm), a nonionic surfactant soluble in water, and performed systematic experiments by gently depositing oil droplets onto its aqueous solutions with Triton concentrations of 0.5, 2, 10, and 75 ×cmc at ambient conditions. The regime map based on the outcomes of the spreading experiments is shown in **Figure** **3**a along with a representative example of the outcome at a fixed concentration (10×cmc) in Figure 3c and the entire dynamic behavior can be observed in Movie S3 (Supporting Information). Results showed that styrene was the only liquid that existed in drop/lens form at all surfactant concentrations. At 0.5×cmc, other liquids formed lens/pseudo‐lens phenomena with a large spreading radius. At 2×cmc, α‐Pinene and dodecane fragmented while others remained in drop/pseudo‐lens form. At 10×cmc, kerosene, and dodecane droplets underwent rapid spreading, followed by near‐complete recoil, then stretched into a thread shape, and ultimately fragmented into smaller droplets. Ethyl butyrate droplet maintained its shape initially but subsequently jumped around on the water‐surfactant (w‐s) surface, oscillated, and spun while simultaneously forming ligaments that fragmented completely. The α‐pinene droplet underwent irregular motion before eventually deforming and fragmenting. At 75×cmc, the strength of the abovementioned phenomenon at 10×cmc was intensified and caused rupturing for all oils, except for styrene, on the water surface. The oil spreading behavior is expected to be indifferent to cooling and this was confirmed by examining the spreading behavior of kerosene and styrene on a cold (10 °C) water‐Triton solution (10×cmc). The rich behavior for each case stems from the competition between various surface tension‐related forces.^[^ ^29^ ^]^ Upon depositing an oil drop at the surfactant‐covered water‐air interface, surfactant molecules promptly diffuse to be absorbed in the newly formed oil‐water interface. However, this absorption is non‐uniform in the initial moments, creating regions with differing interfacial tensions, and stresses leading to destabilization of the interface (Marangoni instabilities) that ultimately causes drops to fragment.^[^ ^30^ ^]^ If such macroscopic behavior extends to condensed oil drops ≈ 𝒪(100 nm), then they too could undergo fragmentation thereby facilitating finer drop formation, but this remains to be studied.

Next, we investigated the self‐emulsification behavior of the oil‐water‐Triton system by suspending pendant drops of Triton solutions in various oils and observing the transformation of the transparent surfactant solution into turbid or opalescent over a 30 min period (See Experimental Section). The results of these experiments are presented in the form of a regime map in Figure 3b where 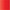 and 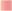 symbolize vigorous and feeble degrees of self‐emulsification respectively. The experiments showed that self‐emulsification was only shown by styrene (at >2×cmc) and ethyl butyrate (only at 75×cmc). Examining the details behind these observations is beyond the scope of this work, but in brief, multiple mechanisms have been proposed behind spontaneous emulsification, including^[^ ^31^ ^]^ interfacial turbulence, diffusion, and stranding, negative interfacial tension, molecule‐mediated pathway, micelle‐mediated pathway, and finally a combination of one or more mechanisms acting together. The molecular pathway is favored for oils with higher solubility such as for styrene and ethyl butyrate (see Table S1, Supporting Information) – but the fact that self‐emulsification was absent at lower surfactant concentrations rules out this mechanism. The most likely explanation for the observed behavior is the micelle‐mediated pathway that is believed to be the primary mechanism for micellar solutions of non‐ionic surfactants, wherein micelles impinge, absorb, and then detach from the oil‐water interface, peeling off some oil with them.

### 2.4 Oil Vapor Condensation on Stagnant Triton X‐100 Solution

In the next phase, we condensed oil vapor on an aqueous solution with fixed Triton concentration (10*×*cmc) held at *T_pel_* = 10 ± 0.5 °C. The video of the top‐down imaging showing the dynamic condensation behavior can be viewed in Movie S2 (Supporting Information) while representative images showing the typical condensation behavior of each oil are shown in Figure 3d. The results indicate that styrene did not form drops on the surfactant solution, but instead formed a milky solution, like 10 and 100*×*cmc cases of *w/o* emulsion (Figure 1a). At 10*×*cmc, styrene condenses as a drop because its *S*_ow(a)_ < 0 (see Figure 3c; Table S2, Supporting Information) in Experimental Section), and at the same time, it also undergoes self‐emulsification induced transfer to the water phase (Figure 3b). Based on these observations, we suggest that the cloaking and submergence mechanism responsible for *w/o* emulsion formation does not play any role herein. Instead, the likely mechanism for emulsion formation is that styrene nano drops nucleate at the *w‐s* surface, and then undergo micellar‐mediated self‐emulsification and transfer within the *w‐s* solution. Condensation provides nanodroplets that, due to their size, can rapidly undergo self‐emulsification. For other oils, micro drops with interference patterns were visible under observation (Figure 3d), suggesting they existed in pseudo‐lens configuration. As indicated before, at 10*×*cmc, other oils spread as films that underwent partial/complete fragmentation (Figure 3c) and did not undergo self‐emulsification (Figure 3b). These observations lead us to conclude that Triton X‐100 is unlikely to form in situ emulsions via condensation without agitation of the flowing oil. However, it is entirely possible that for other surfactants or surfactant mixtures, the combination of condensation, spreading dynamics, and self‐emulsification phenomena could result in nanoscale emulsions for a variety of oils. Future studies can explore these aspects.

### 2.5 Continuous Synthesis of *o/w* Emulsions by Condensation on Flowing *w‐s* Solution

**Figure 4:**
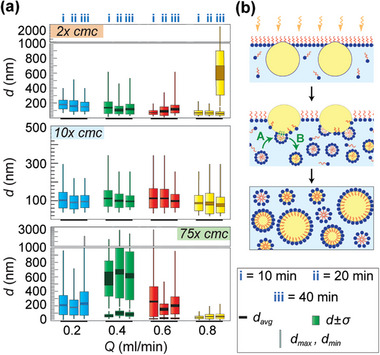
a) Bar chart of emulsion sizes as a function of the surfactant concentration, w‐s flow rate, and condensation time. Styrene was heated to ≈30 °C for vaporization and then condensed on a flowing water‐surfactant solution held at 10 ± 0.5 °C. b) Proposed schematic explaining the formation of nanoscale‐sized emulsions through the micellar‐mediated pathway.^[^ ^15^ ^]^

Since styrene showed the clearest signs of in situ emulsion formation, we next sought a continuous synthesis of its emulsion in a similar vein as done for water‐in‐oil emulsions before. Before proceeding with the continuous synthesis, we conducted separate experiments to examine the styrene spreading and styrene vapor condensation on the stagnant *w‐s* solutions (2, 10, and 75 *×*cmc) and confirmed that they were all similar. Next, we modified our continuous flow setup shown in Figure 1d to condense styrene on *w‐s* solutions of various Triton concentrations (2, 10, and 75 *×*cmc) flowing within the groove at flow rate *Q* ranging from 0.2 to 0.8 mL min^−1^ (see Figure S6, Supporting Information). The setup was held at *T_pel_* = 10 ± 0.5 °C for different periods (*t_con_* = 10, 20, and 40 min), and the resulting emulsified solution dripping from the outlet hole was collected in a beaker pre‐filled with a small volume of the respective *w‐s* solution. At the end of the *t_con_*, the solution was promptly taken to DLS for size analysis. The results from these experiments for different surfactant concentrations, condensation times, and *w‐s* solution flow rates are comprehensively reported in **Figure** **4**a. The raw DLS data is shown in Figure S7 (Supporting Information) along with pictures of collected emulsions. The results show that in most cases, the emulsion was highly monodispersed, and drop sizes were in the nanometer range irrespective of how long vapor was condensed on the solutions. At 2*×*cmc, the average droplet diameter was ≈150 nm for *Q* = 0.2–0.6 mL min^−1^, but at *Q* = 0.8 mL min^−1^, a second peak appeared when vapor was condensed for a long period (*t_con_* = 40 min). At 10*×*cmc, the average droplet diameter was ≈100 nm for all the flow rates, regardless of how long the condensation was done. Surprisingly, at 75*×*cmc, the droplet sizes were larger, and more polydisperse than in the preceding cases. At *Q* = 0.4 mL min^−1^, we saw two peaks, the first with an average diameter of ≈100 nm and monodisperse, and the second more polydisperse peak with an average diameter of 600 nm. At *Q* = 0.6 mL min^−1^, the first peak overlapped with the micelle sizes, and the second peak with an average drop diameter of ≈200 nm was observed. Finally, at *Q* = 0.8 mL min^−1^, the collected solution showed size distribution completely overlapping the micellar sizes. As discussed in the previous section, for systems with nonionic amphiphiles, a micelle‐mediated pathway is more likely because their micelles can come very near the drops owing to the absence of electrical repulsion. Figure 4b shows that styrene nano drops nucleate at the w‐s surface, then undergo micellar‐mediated self‐emulsification and transfer within the *w‐s* solution to form the emulsion. This process involves micelles impinging at the oil/water interface (Step A), following that they take up the oil molecules either by adsorption or by dissociation into surfactant monomers that adsorb at the oil‐water interface (Step B). Efforts to study the extended stability of these emulsions were hindered by the evaporation of styrene out of the emulsion due to its volatility (see Figure S8, Supporting Information).

### 2.6 Outlook

Our study focused on overcoming key obstacles for EVC adoption by showing a new route for large‐scale emulsion production while shedding light on all relevant aspects related to it at the cross‐section of interfacial science, multiphase heat transfer, and surfactant dynamics. Conventional techniques like sonication typically synthesize emulsion in batches and generate excess heat, limiting their use with flammable liquids, and heat‐sensitive chemicals (Figure S9, Supporting Information). In their comparison, EVC yields continuous production of w/o and o/w emulsions with smaller droplet sizes (Figure S9, Supporting Information) without any heat generation. However, despite the advantages of EVC and the advancements reported herein, several challenges remain ahead. For example, methods to incorporate additives in condensed drops remain to be explored. A potential solution to overcome this challenge is modifying EVC to create multiple emulsions, for example, by collecting the o/w emulsion in another oil (Figure S10, Supporting Information), and further expanding the possibilities by combining EVC with coflowing systems or microfluidics. Accurately measuring the dispersed phase of o/w emulsions by EVC is also a challenge as oil condensation rates are a strong function of thermodynamic conditions and need further studies. The role played by aqueous surfactants on oil spreading can complicate the formation of nanoscale o/w emulsions but can potentially be addressed by incorporating co‐surfactants or integrating EVC with a small degree of homogenization. Finally, while emulsions by vapor condensation remain in nanoscale sizes over short periods, further studies are needed to study the extended stability of emulsions over weeks/months. Addressing the remaining challenges will be crucial in realizing the full potential of EVC technology, paving the way for its widespread adoption across various industries, and shaping the future of this promising method.

## 3 Conclusion

In conclusion, a new method for continuously formulating *w/o* and *o/w* monodisperse nano‐sized emulsions has been developed by introducing vapor condensation over unidirectionally flowing liquid‐surfactant solutions. The size and monodispersity of the emulsions remained relatively constant over time at all tested flow rates. We find that the spreading kinetics and self‐emulsification of oil‐water‐surfactant systems play a crucial role in determining the stability of the condensed droplets. We find that self‐emulsification is possible for certain oil‐water‐surfactant combinations, and this could aid the formation of EVCs. Overall, our study provides a framework for designing efficient and stable EVC systems by controlling the flow rate of the oil‐surfactant solution and carefully selecting the appropriate surfactant and oil combinations. Our findings represent an important step toward guiding the potential applications of the vapor condensation method for emulsions and colloidal systems including drug delivery, cosmetics, and food science.

## Conflict of Interest

The authors declare no conflict of interest.

## Supporting information

Supporting Information

Supplemental Movie 1

Supplemental Movie 2

Supplemental Movie 3

## Data Availability

The data that support the findings of this study are available from the corresponding author upon reasonable request.
