# Supplementary material for: Continuous Synthesis of Nanoscale Emulsions by Vapor Condensation (EVC)
Source: Adv Sci (Weinh). 2024 Feb 14;11(15):2307443. doi: 10.1002/advs.202307443 (PMC11022740; doi:10.1002/advs.202307443)
Supplement: Supplementary file 1 — Supporting Information [file ADVS-11-2307443-s001.pdf]

ADVANCED SCIENCE
Open Access

Supporting Information

for *Adv. Sci.*, DOI 10.1002/advs.202307443

Continuous Synthesis of Nanoscale Emulsions by Vapor Condensation (EVC)

Sushant Anand, Vincent Galavan and Mahesh Uttamrao Mulik*

Continuous Synthesis of Nanoscale Emulsions by Vapor Condensation (EVC)

Sushant Anand[1], Vincent Galavan[1,2], Mahesh Uttamrao Mulik[1,3]*

[1] Department of Mechanical and Industrial Engineering, University of Illinois at Chicago, USA
[2] Department of Nuclear Science & Engineering, Massachusetts Institute of Technology, USA
[3] Spruce Up, India

Email: sushant@uic.edu (S.A.)

1. Materials and Methods

Liquids used in the current study. All chemicals were purchased from Sigma-Aldrich and used without further purification. The chosen oils' quality was anhydrous with purity≥99% for dodecane and ethyl butyrate, or purity≥98% for α-Pinene and reagent grade with purity≥95% for kerosene and styrene. The properties of oils are given in **Table S1**. To prepare water-in-oil emulsions, non-ionic surfactant Span-80 ($C_{24}H_{44}O_6$, Sorbian monooleate, HLB=4.3, MW=428.61 g/mol, *cmc*=0.2 mM; Γ_∞=4.42×10^{-6} mol/m^2 and κ=10 m^3/mol for water-hydrocarbon[1]) was used. To prepare oil-in-water emulsions, a non-ionic surfactant Triton X-100 (Octylphenol ethoxylate, HLB=13.5, MW=625 g/mol, *cmc*=0.24 mM, cloud point=66 ºC; Γ_∞= 1.15×10^{-6} mol/m^2 and κ=5050 m^3/mol for water-hydrocarbon[1]) was used. In the above description, HLB, MW, Γ_∞, κ, and *cmc* stand for hydrophilic-lipophilic balance, molecular weight, maximum surfactant interfacial coverage, Langmuir constant, and critical micelle concentration, respectively.

	Styrene	Dodecane	Kerosene	α-Pinene	Ethyl Butyrate
**Structure**	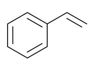	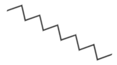		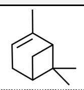	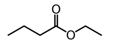
ρ_o (kg/m^3)[2]	906	748.7	810	860	878.5
n_o[2]	1.547	1.42	1.44	1.465	1.389
μ_o ($mPa\cdot s$) [2]	0.7	1.485	1.64	2.11	0.64
T_m (^oC) [2]	-30.6	-9.6	-25	-64	-98
T_b (^oC) [2]	145	216	150-300	155	121
$Sol_{o/w}$ (ppm)	321.6 [3]	0.004 [3]	5-10	2.5	6168 [3]
P_v ($mmHg$)[2]	4.442	0.13	0.23	5.3	11.3
γ_{oa} (mN/m)	23.31	25.4 ± 0.5[4]	25.2 [4]	25.87 [5]	24.46 [6]
γ_{wa} (mN/m)	72.2	72.8 ± 0.5	72.8 ± 0.5	72.8± 0.5	32.58 [6]
γ_{ow} (mN/m)	22.88	52.8 ± 1 [4]	34.7 ± 1 [4]	18.9 [5]	15.7 [6]
$S_{ow(a)}$ (mN/m)	25.6	−6.4±1.2 [4]	12.3±1.2[4]	27.4	-7.58 [6]
$S_{wo(a)}$ (mN/m)	-71.4	-100.6 [6]	-82	-65	-23.8 [6]

Table S1| Oil properties at room temperatures. Here ρ_o, n_o, γ_{oa}, μ_o, P_v, T_m, T_b denote density, refractive index, surface tension, dynamic viscosity, vapor pressure, melting point, and boiling point of the oil respectively. $Sol_{o/w}$ denotes the solubility of the oil-in-water. The interfacial tension between water and oil was measured using drop-shape analysis (OCA 15 Pro, Dataphysics) in room temperature conditions. Based on interfacial tension, the spreading coefficient of oil on water is defined as $S_{ow(a)} = \gamma_{wa} - \gamma_{ow} - \gamma_{oa}$.

Surfactant solution preparation. Oil-surfactant solutions at different concentrations (10× and 100× *cmc*) were prepared by mixing the surfactant (Span-80) with the oil (kerosene) and sonicating the solution for an hour. For the preparation of water-based surfactant mixtures, different solutions were prepared for 0.5, 2, 10, and 75 ×*cmc* of respective surfactant (Triton X-100) in ultrapure water. The mixtures were thoroughly mixed using either a bath-type or horn ultrasonicator for an hour at room temperature.

Interfacial tension measurements. The interfacial tension between water and oil in the presence of surfactants was measured using the pendant drop technique in a goniometer (OCA 15 Pro, Dataphysics) in room temperature conditions. For Span-80, a water droplet was slowly injected inside the kerosene-surfactant solution stored in a quartz cuvette, whereas for Triton X-100, the *w-s* solution was injected in the pure oils held within the quartz cuvette. In all the cases, dynamic interfacial measurements were performed for different oil-water-surfactant combinations and the final interfacial tension was identified by locating the steady-state values. Example measurements of surface tension for a surfactant (Triton X-100) and spreading coefficient are given in **Table S2** below.

	Water	Styrene		Dodecane		Kerosene		α-Pinene		Ethyl Butyrate	
**Conc.**	γ_{wa}	γ_{ow}	$S_{ow(a)}$	γ_{ow}	$S_{ow(a)}$	γ_{ow}	$S_{ow(a)}$	γ_{ow}	$S_{ow(a)}$	γ_{ow}	$S_{ow(a)}$
0.5×*cmc*	41	21.0	-3	6.0	9	30.0	-14	12	-37	13	2.6
2×*cmc*	29.9	20.2	-14	2.9	1.4	2.4	2.5	2	-27	5.3	-1.3
10×*cmc*	30	14.6	-8	4.0	0.4	2.4	2.6	2	-27	5.3	-1.2
75×*cmc*	31	12.5	-5	2.6	2.8	2.4	3.6	2	-27	5.3	-0.2

Table S2| Measured interfacial tension, γ and oil spreading coefficient on water, $S_{ow(a)}$ in presence of Triton X-100. Units: mN/m.

Emulsion characterization. In all the cases, the emulsion collected was promptly analyzed at room temperature using the Malvern Zetasizer Nano dynamic light scattering (DLS) instrument. The experiment entailed conducting 12 runs for each measurement lasting 10 seconds, analyzing samples following a 2-minute equilibration at 25°C, and presenting the outcomes as the mean of three measurements. Each sample's correlation function is analyzed via cumulant analysis to determine the average emulsion diameter and polydispersity.

Water condensation on stagnant oil. The setup described in a previous manuscript[4] was used. Experiments were performed in a closed chamber to minimize any convection effects. The copper container (inner diameter 5.5 cm) with PTFE walls was cooled to 2 °C (air temperature T_{air} =23°C, RH~70%, dew point T_{dp} ~17 °C), and condensation on the oil was observed using a Zeiss stereo microscope (Axio Zoom.V16) with videos recorded using Nikon D810 camera at 30 fps.

Water condensation on flowing oil solution. A special setup was fabricated for this purpose (Figure 1d). An aluminum plate with a semi-cylinder groove (5 mm width, 140 mm length, and 2 mm depth) was attached to a PTFE plate (with a slit of similar width and length as an aluminum plate). PTFE plate inhibited condensation around groove edges while also preventing the solution overflow in the groove. One end of the groove had an outlet hole with an attached needle (2 mm diameter) for collecting the solution. The oil-surfactant solution was filled in a 50 ml Hamilton syringe connected with a syringe pump through which the flow rate was adjusted. After placing the aluminum-PTFE plate on the Peltier, the oil-surfactant solution was injected on one end of the groove (opposite to the exit hole) using a needle attached to the syringe. Once the steady flow conditions were reached, the Peltier was switched on to cool the flowing solution. As the temperature reached below the dew point (T_{dp}), drops condensed on the flowing solution forming an emulsion. The emulsion from the collection needle was collected in a beaker with a magnetic stirring bar (rotating at 400 rpm) to mix and homogenize the emulsion. To prevent potential destabilization of ejected emulsion drops upon impact with a wettable surface like glass, the beaker was pre-filled with a small quantity of the same oil-surfactant solution that was used during an experimental run. The entire setup was placed in a glovebox and experiments were performed under the same conditions as described for the stagnant solution above.

Oil spreading on *w-s* solution. We filled a glass petri dish with a *w-s* solution and illuminated it from the bottom using a cold-light source to prevent heating. Using a syringe pump, 10 µl oil drops stained with Oil Red O dye were gently deposited onto the 8 ml *w-s* solution using a 0.4 mm diameter needle. The spreading behavior was monitored by using a vertically mounted camera (Point Grey Flea®3 USB 3.0) attached to a long-distance lens (Infinity lens) at 30 fps.

Oil vapor condensation on stagnant *w-s* solution. A special setup was fabricated to vaporize the oil and visualize its condensation behavior on surfactant solutions (Figure S5). A copper container for holding and cooling the aqueous solutions was held within a PTFE enclosure. A second copper cylinder with an embedded groove was used for holding oil and vaporizing it with the help of an externally wrapped flexible heater. The two copper containers were separated from each other using a second PTFE enclosure placed on the first PTFE enclosure. A thermocouple type (K) was attached to the oil container to monitor its temperature and help keep it constant during the experiment. A special glass window attached to the second container was used to visualize oil condensation dynamics using the microscope (Axio Zoom.V16). After loading the first copper container with *w-s* solution and the second copper container with oil, the assembled setup was placed on the Peltier. The assembled setup provided a seal-proof way to prevent the oil vapors from escaping outside and helped maintain the oil vapor in saturated condition within the chamber. Since one of the tested oils (styrene) is carcinogenic, strict environmental control was undertaken to prevent the escape of its vapors into the environment and all experiments were done in a chemical fume hood. The Peltier temperature was lowered to 10°C while the flexible heater temperature was set to 30°C. The 20°C temperature differential allowed for oils to condense on the cold *w-s* solutions. The condensation process was recorded using the Nikon D810 camera at 30 fps.

Oil vapor condensation on flowing *w-s* solution. A special setup was fabricated for this purpose (Figure S6). An aluminum base plate with an overlaid PTFE plate with similar geometrical features as discussed previously for *w/o* continuous synthesis was fabricated. A thin-walled hollow cuboid of copper with embedded grooves was prepared to act as an oil holder and was wrapped externally with a flexible heater. This was placed on the PTFE plate to separate the heater and condenser sections. A copper cover with a glass window was assembled on top of the oil holder to prevent the evaporated oil from escaping the chamber and keep the condition within the chamber saturated. The surfactant solution was injected into the aluminum groove through the injection needle (connected to a syringe and syringe pump) and the resulting emulsion was collected using a beaker placed below the end of the groove fitted with a collection needle. The volume flow rate of *w-s* solution within the aluminum groove is adjusted by the syringe pump and varies from 0.1 to 0.8 ml/m.

Nucleation rate calculations. The volumetric nucleation rate ($m^{-3}s^{-1}$) is given by $J_{hom} = Zf^*C_0\exp[-W^*/kT]$ where Z is the Zeldovich non-equilibrium factor, f^* is the collision rate of monomers with the critical cluster or the attachment frequency, C_0 is the concentration of nucleation sites that is equal to a total number of molecules present

in an environment, W^* is the work of formation ($W^*=4\beta^3/27\alpha^2$) of critical cluster size n^* where $n^* = (2\beta/3\alpha)^3$. Here, $\alpha = kT\ln(SR)$ and $\beta = \psi\pi\left(\frac{3v_m}{\pi\psi}\right)^{2/3}\gamma$ where γ is interfacial tension of drop-air interface.[7] The Zeldovich factor is given by $Z = \frac{3\alpha^2}{4\beta^2}\sqrt{\frac{\beta}{\pi kT}}$ and f^* is given by[7] $f^* = (48\pi^2 v_m n^*)^{1/3}\alpha_m D_{ab} SR \times C_s$ where D_{ab} is the mutual diffusion coefficient of the vapor in the solvent medium, and SR is the saturation ratio defined as $SR=C_c/C_s$ where C_c is the solute concentration and C_s is the solubility of the solute molecule in the solvent, and α_m is the monomer sticking coefficient (accommodation coefficient).[7a, 8] The shape factor ψ is related to the lens angles of a drop on a non-cloaking liquid and formulation for computing it is given in Anand et al.[7b] The heterogeneous nucleation rate J_{het} ($m^{-2}s^{-1}$) is obtained by $J = J_{hom} \times \left(Volume_{drop}/Area_{drop}\right)$. For further details on nucleation theory for heterogeneous nucleation, see works by Beysens[9] and Christian[10]. For our experimental conditions (peltier temperature, humidity, and air temperature), the resulting supersaturation was ~1.8. Using above equations, and entering the relevant values of the water molecule diameter (3.93 Å) and D_{ab} (2.5×10^{-5} m^2/s), we find that $J \sim 10^{16}$ $m^{-2}s^{-1}$ for dodecane (whose lens angles can be computed from knowledge of interfacial tension values[7b]), and for kerosene, $J \sim 10^{17}$ $m^{-2}s^{-1}$.

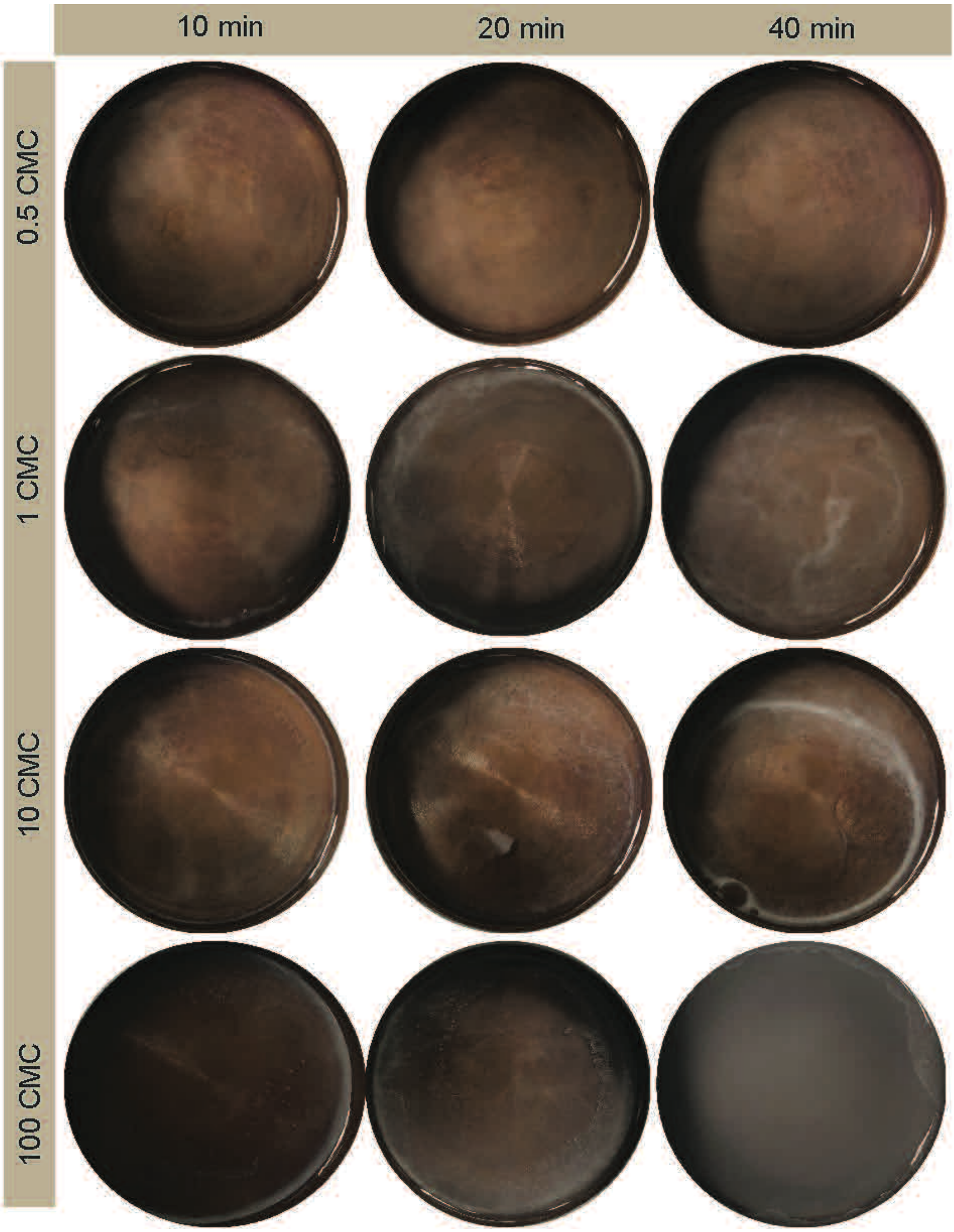

Figure S1. Top-view images of macroscopic condensation and emulsion formation on confined oil (i.e. zero flow rate of oil) as a function of surfactant concentration and condensation time (t_{con}).

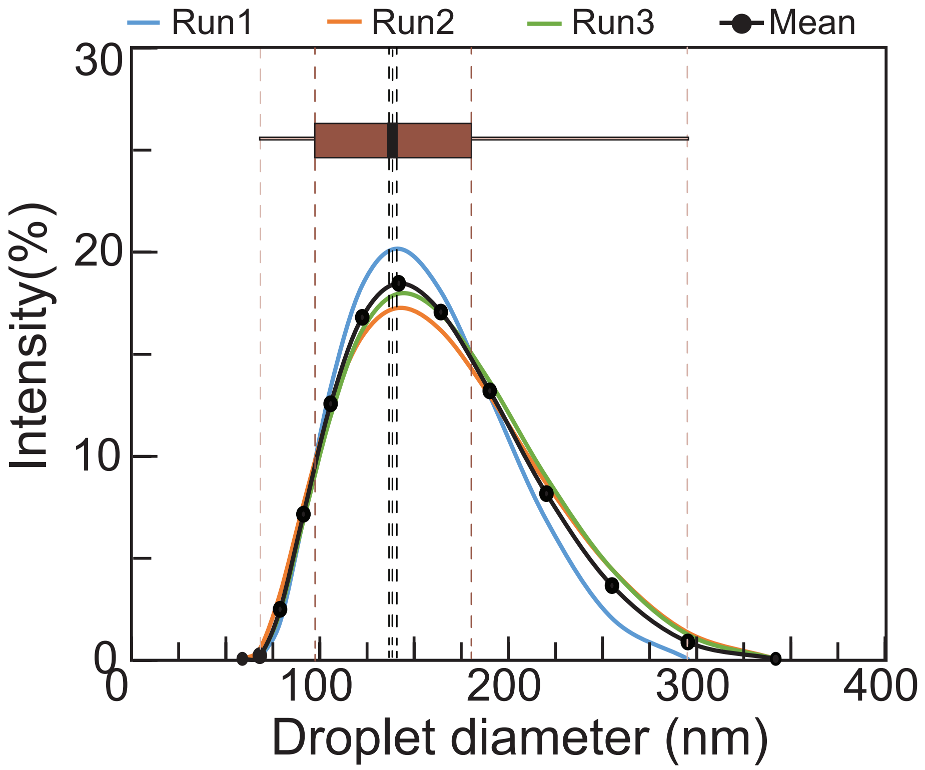

Figure S2. A guide to interpreting DLS measurement (mean, standard deviation, minimum and maximum) of emulsion droplet size: (oil phase: Kerosene with 100cmc Span 80, condensation time: 40 min and RH=70%) for three consecutive DLS runs. DLS measurement of micelle size at ~200 X cmc. The peak shows micelle size is around 120nm.

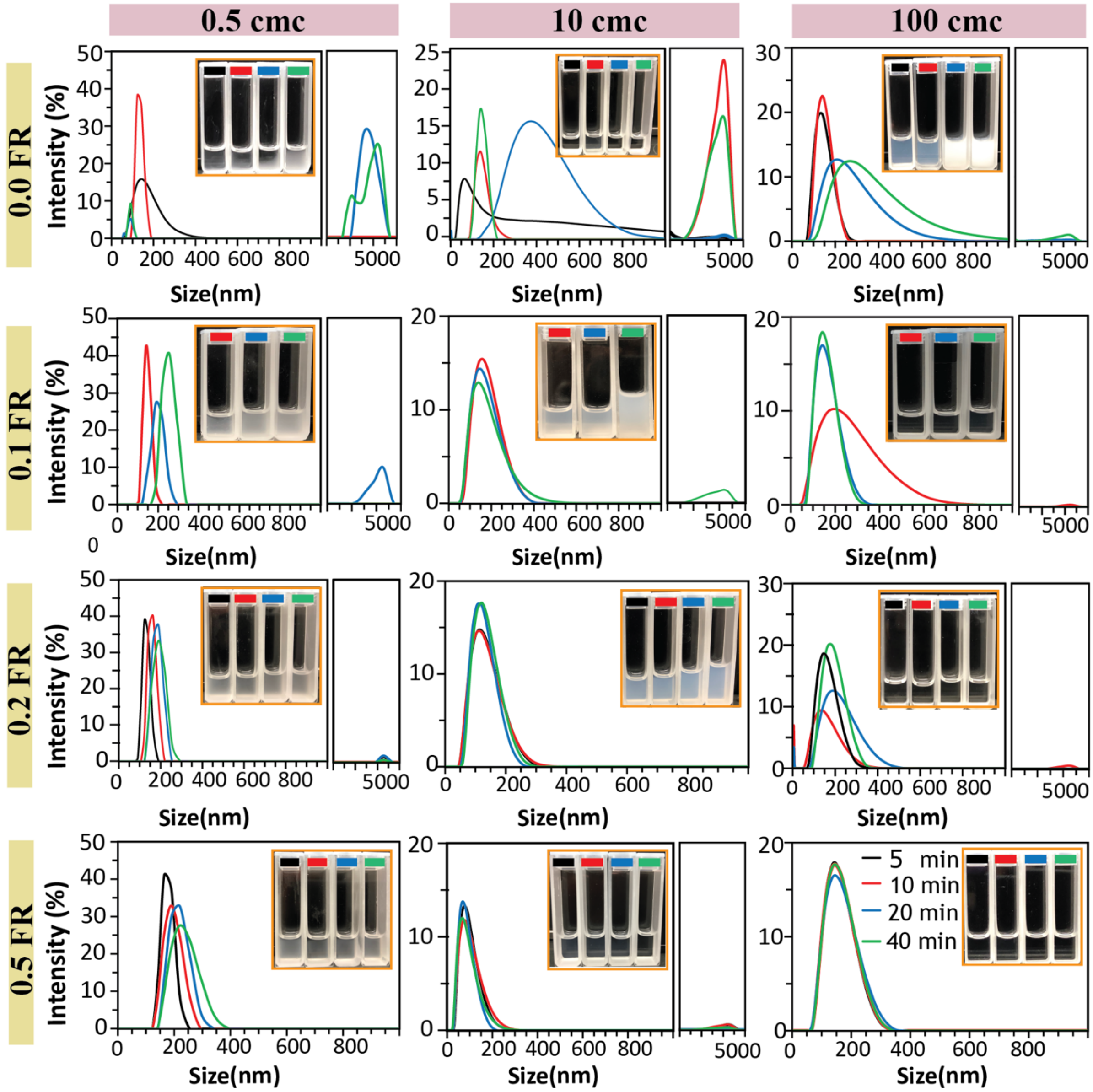

Figure S3. **DLS size distribution and optical images of water in oil emulsions obtained by continuous synthesis approach as a function of surfactant concentration, condensation time, and flow rate.** Increasing the surfactant concentration eliminates the second peak depicting the emulsion size (<1 µm) even at zero-flow rate. Images indicate that the emulsion turbidity alters by increasing the flow rate, passing from milky to somewhat cloudy and transparent, respectively as the oil flow rate is increased. The emulsion size is found to decrease with an increase in flow rate owing to the rapid availability of fresh (i.e. bare) oil-air surface for droplet nucleation and a decrease in the crowding of condensed water droplets – factors that decrease the likelihood of coalescence between them and consequently suppress the growth of emulsion droplets. In all experiments, the dew point (T_{dp}) was ~17±1 °C, and Peltier temperature (T_{pel}) was 2±0.5 °C.

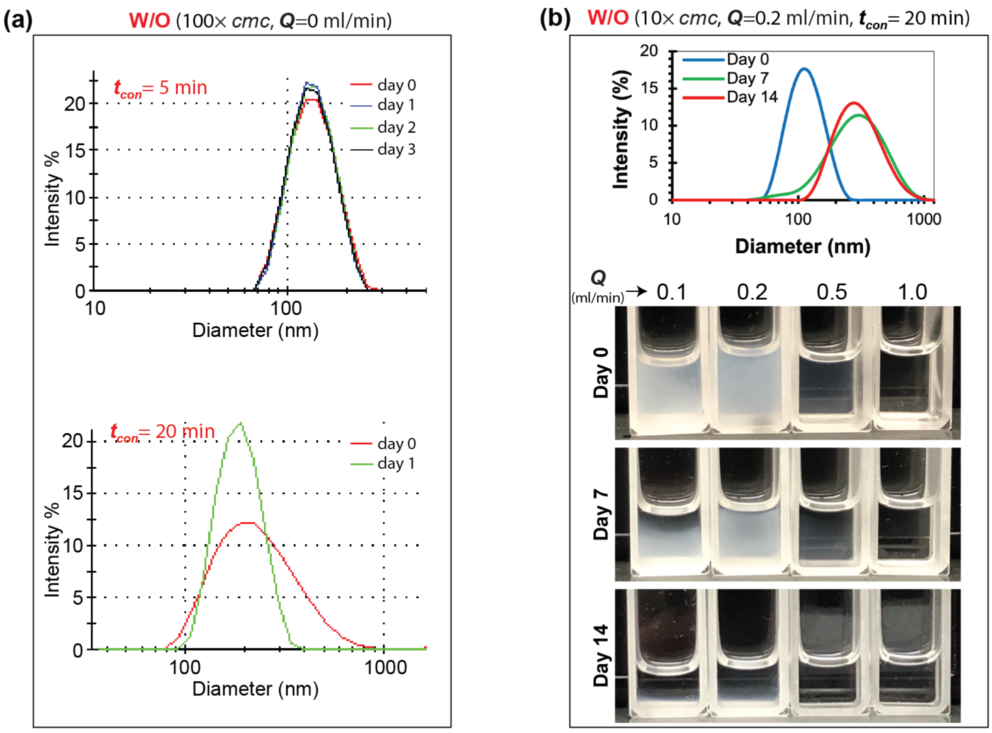

Figure S4: (a) Size of w/o emulsion prepared on stagnant Kerosene oil with 100×*cmc* Span-80. Top row graph shows the evolution over three days of emulsion prepared by condensing water vapor over 5 mins. The emulsion size remains invariable over three days. Bottom row shows the evolution over a day of emulsion prepared by condensing water vapor over 20 mins. The emulsion monodispersity increases in a day. The larger size measured on Day-0 may be related to emulsion drops forming aggregates because of the crowding of condensed drops at the oil drop. These may have become dispersed over a day leading to a decrease in polydispersity. **(b) Size of w/o emulsion prepared on flowing solution of Kerosene oil with 10×*cmc* Span-80 as observed over a period of two weeks.** The bottom row images show changes in the visual appearance of emulsions prepared with 10×*cmc* Span-80 over different flow rates. The emulsion polydispersity increased from Day-0 to Day-7 but thereafter remained stable. The increase may be related to the lower amount of surfactant in this case. In all experiments, the dew point (T_{dp}) was ~17±1 °C, and the Peltier temperature (T_{pel}) was 2±0.5 °C.

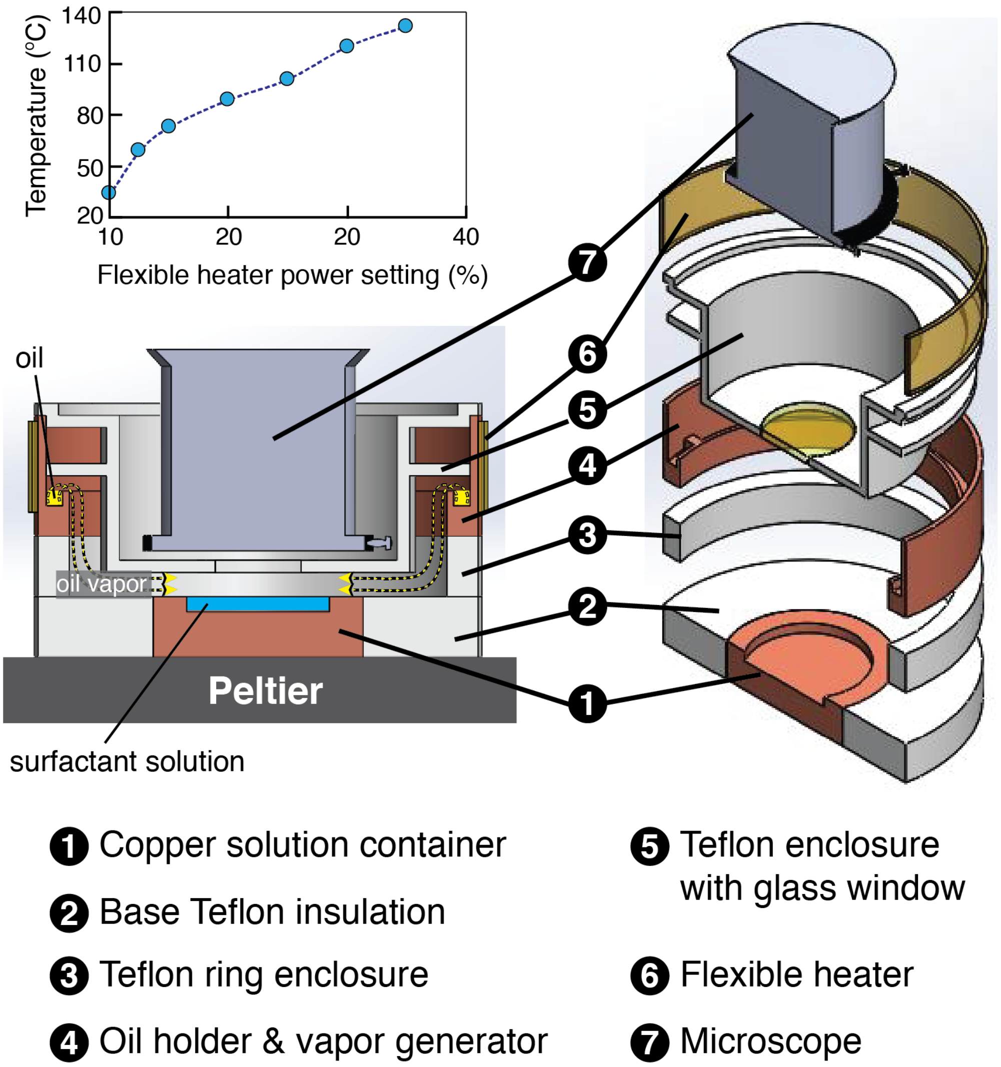

Figure S5. Schematic of oil-in-water condensation flow setup for the stagnant solution. The setup consists of a circular copper container (Part. 1) placed on a Peltier device and surrounded by a Teflon insulator (Part. 2) to assemble other components. Part 1 has an inner and outer diameter of 50 mm and 75 mm respectively, and depth of 5 mm to hold the surfactant solution. Part 2 height is 20 mm, and its inner diameter is 75 mm which snugly fits Part 1, and its outer diameter is 148 mm. Part 3 (Teflon enclosure) has inner and outer diameters of 128 mm and 148 mm respectively and has a height of 20 mm. An oil container (Part. 4), which is a thin-walled hollow cylinder with a groove embedded inside its bottom, is wrapped by a flexible heater (Part. 6) and attached with a K-type thermocouple to keep the oil temperature constant during the experiment. Part 4 height is 40 mm, its inside and outside diameters are 128 mm and 148 mm respectively, and the groove for holding the oil has depth of 5 mm. A Teflon ring enclosure (Part. 3) is placed on the base Teflon to separate the hot oil container from the Peltier device, and a Teflon enclosure (Part. 5) is placed on the oil container to prevent oil vapors from escaping. The Teflon enclosure has a total depth of 56 mm, and its inner diameter of 96 mm provides sufficient room for the microscope lens (Part. 7) to move up and down during the recording process. It has a glass window of a diameter 40 mm for visualization.

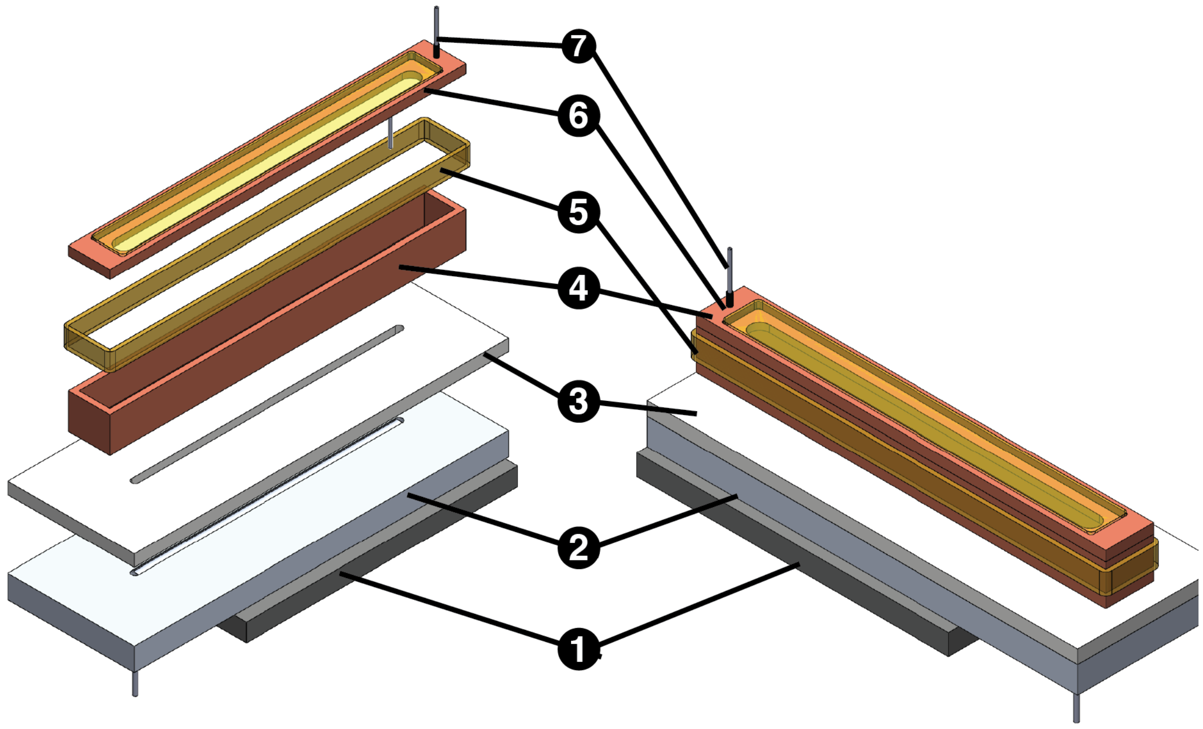

❶ Peltier

❷ Aluminum plate with groove

❸ Base Teflon insulation

❹ Oil holder, vapor generator and enclosure

❺ Flexible heater

❻ Copper cover with glass window

❼ Needle for surfactant solution inlet

Figure S6. Schematic of flow setup for continuous emulsion synthesis. The injection needle delivers the surfactant solution into the Aluminum groove, which is attached to the Peltier at 10°C. The surfactant solution gradually fills up the groove, and its temperature decreases. The flexible heater is set at 30°C, causing the oil inside the container to evaporate. The vapors pass through the Teflon insulator gap and condense as oil droplets on the cooled water-surfactant solution. The resulting emulsion is collected in the beaker and mixed using a stirring magnet rotating at 400 rpm. The fate of the oil droplets is determined by the surfactant type and concentration.

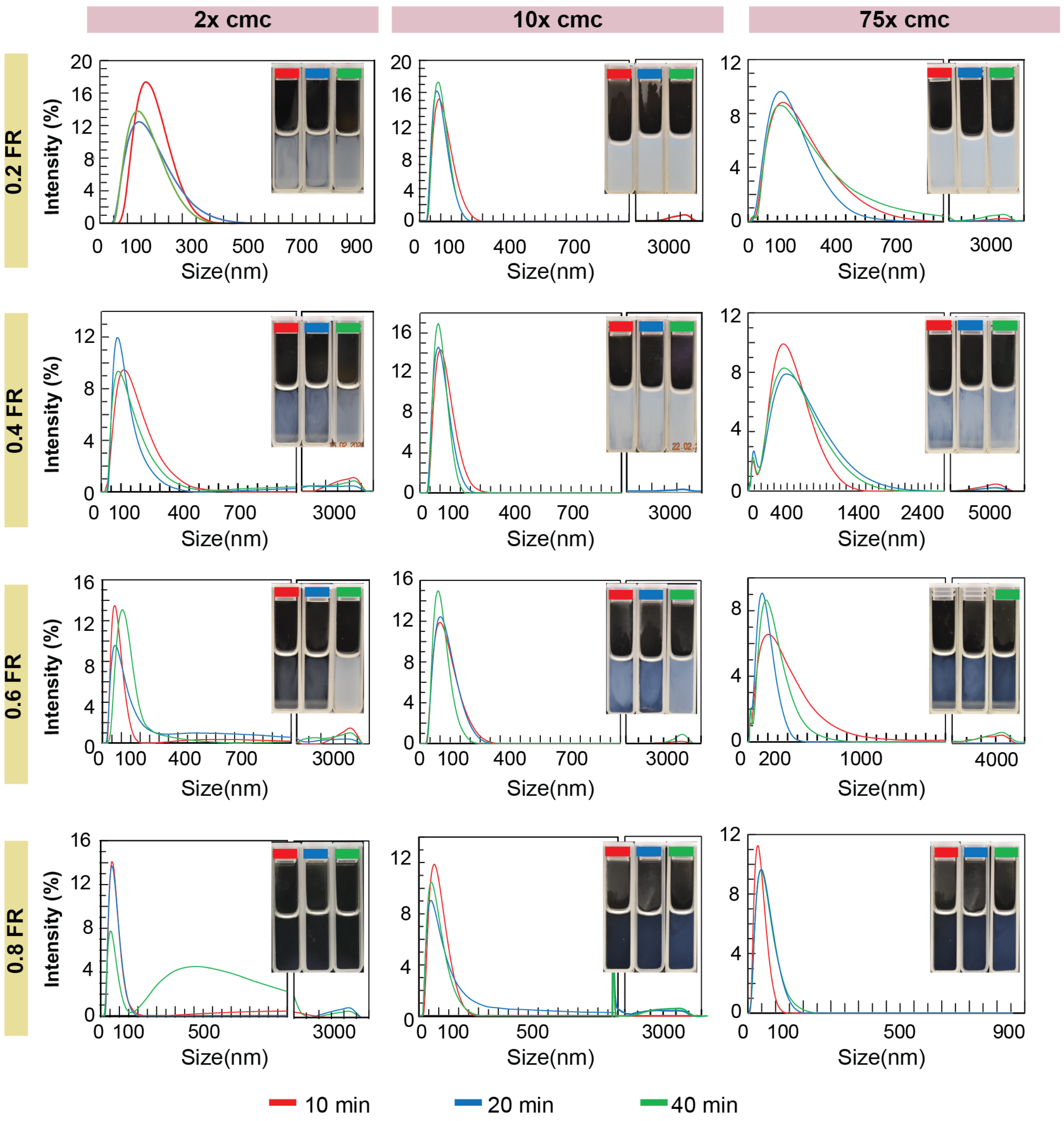

Figure S7. DLS measurements and optical images of oil in water emulsion made by continuous synthesis approach at different condensation times, flow rates, and surfactant concentrations. The photos reveal that using higher surfactant concentration results in a denser milky emulsion, which its color fades by increasing the flow rate (like water in the oil case). The results depict that at moderate flow rates (~0.2 to 0.4 ml/min), the obtained emulsions have narrower size when the droplets experience a fragmentation or suppressed spreading mode ($c <$ 10 *cmc*) rather than rupturing mechanism ($c = 75 \times cmc$). Almost all the obtained emulsion sizes, regardless of the performance parameters, are less than 1 µm (a second distribution greater than 1 µm is seen for some cases with negligible intensity compared to the main peak size). In all experiments, the water surfactant solution was held at 10±0.5 °C, and oil was heated to ~ 30 °C for vaporization.

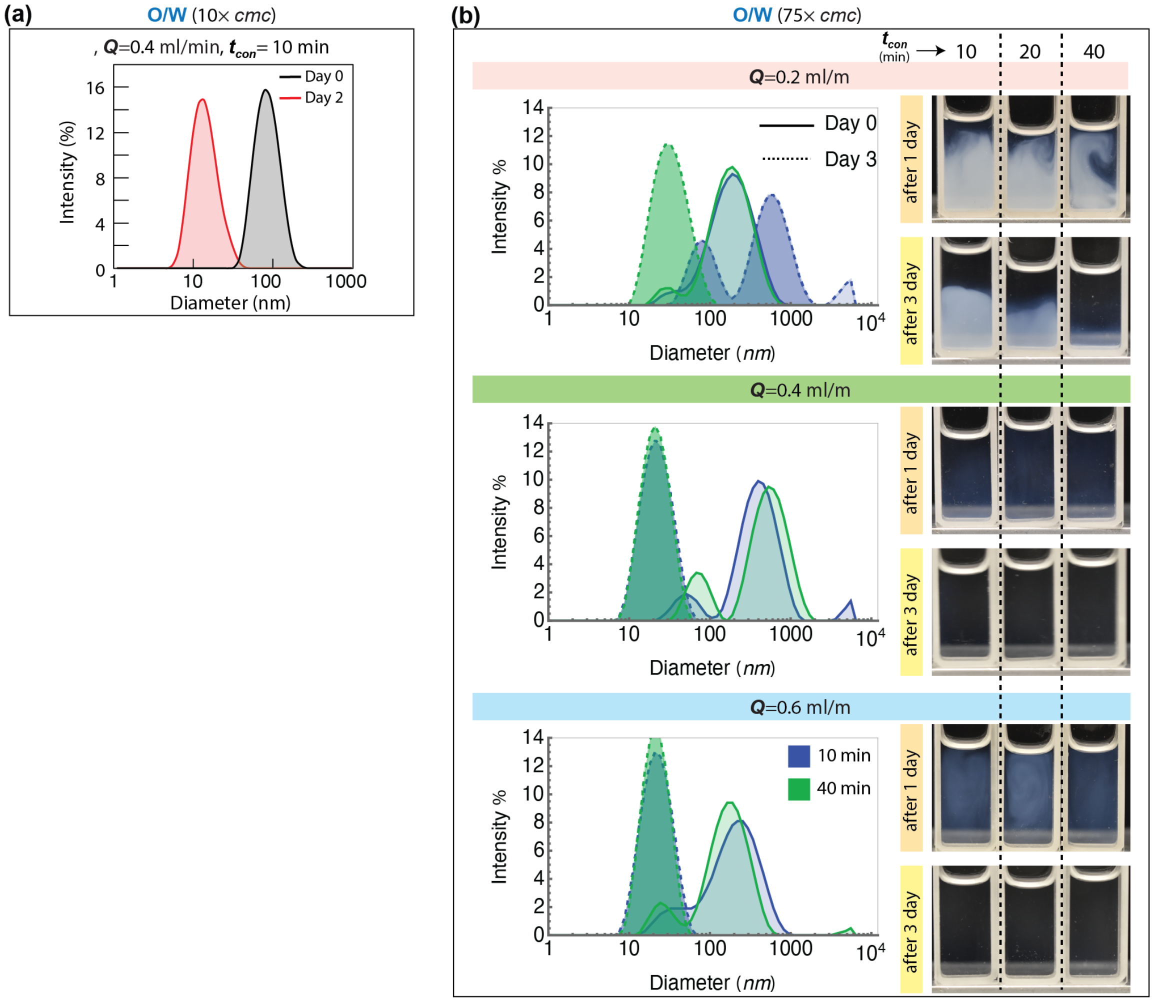

Figure S8: (a) Size of o/w emulsion prepared by condensing styrene for 10 mins over 10×*cmc* water-Triton solution flowing at 0.4 ml/min. Emulsion sizes were measured on the day of their formation and the next day. Within two days, the average emulsion size had decreased from 100 nm to ~11 nm (similar to the typical micelle size reported for Triton X-100) for the case of styrene condensing over a period of 40 mins. These changes may be related to styrene's volatility likely resulting in it evaporating from the water-surfactant solution. For the case of styrene condensing over 10 mins, the emulsion size increased over three days – suggesting a possible role played by Ostwald Ripening. **(b) Size of o/w emulsion prepared by condensing styrene for 10 mins and 40 mins over 75×*cmc* water-Triton solution flowing at different flow rates**. As observed in (a), the emulsion size decreased to ~10 nm, and the solutions became increasingly transparent over time – likely due to styrene's volatility. In all experiments, the water surfactant solution was held at 10±0.5 °C and oil was heated to ~ 30 °C for vaporization.

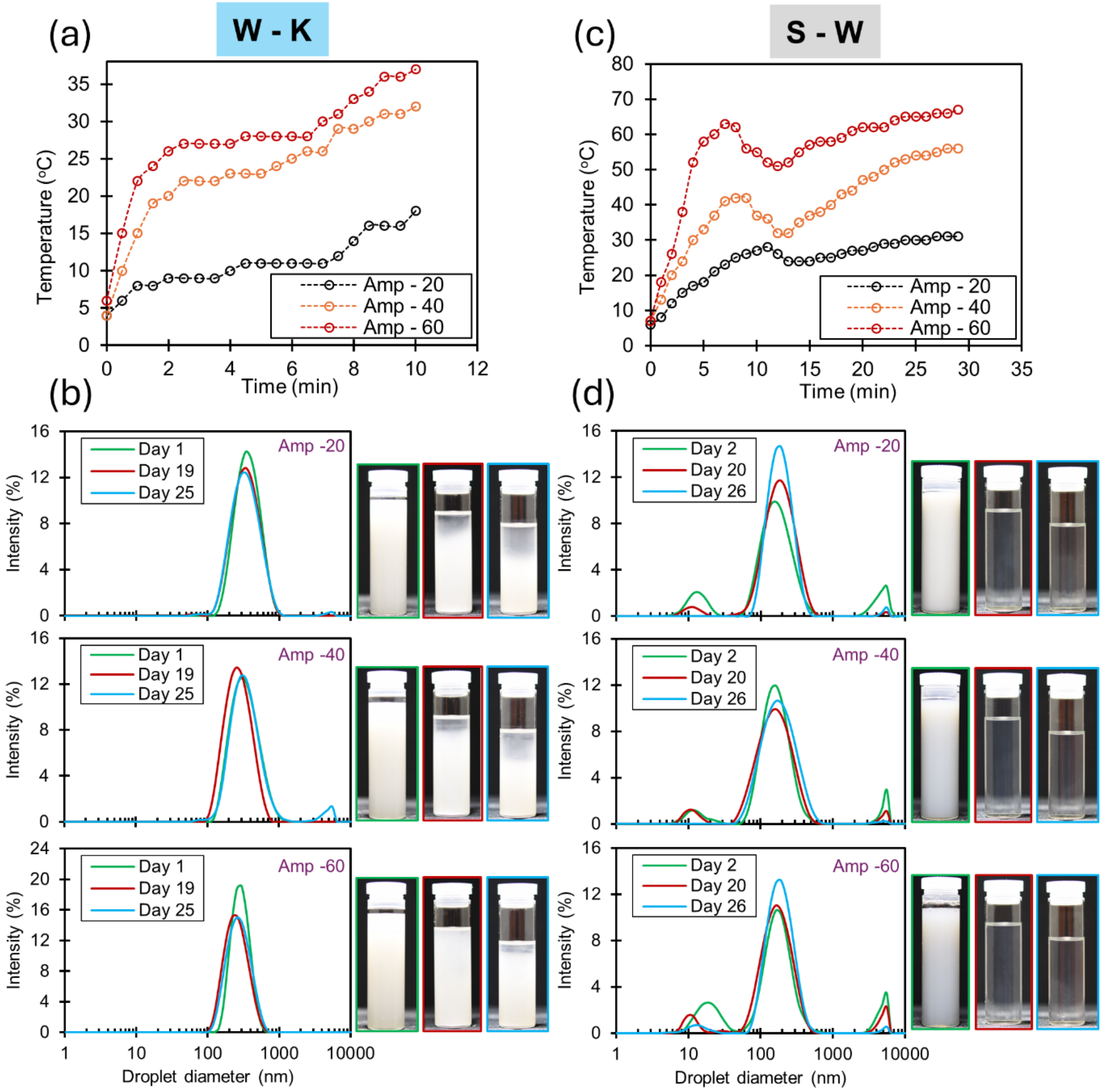

Figure S9| Emulsions by sonication. We manually mixed surfactants (10× *cmc*) and 1% wt. of the dispersed phase in a glass beaker, then placed the resulting solution inside a larger beaker with ice in the gap. We attached a K-type thermocouple to the inner beaker to monitor temperature during 40-minute ultrasonication (Qsonica Q700) at 20%, 40%, and 60% power to emulsify solutions. The temperature rise and flash point of the continuous phase limit the sonication time. **(a)** Temperature increase of water in kerosene (with Span-80 surfactant) emulsion solution at three power settings. Without ice, the emulsion exceeded 80°C in 5 minutes at 40% power, posing a danger for further operation. **(b)** The DLS measurements show the size distribution of W/O emulsions for the same composition across three power settings. Corresponding high-resolution images are presented for reference. For similar solution composition, EVC produced smaller initial droplets (100 nm), growing to ~300 nm in a week as discussed in **Figure S4b**, whereas sonication produced stable ~300 nm droplets over three weeks. **(c)** Temperature rise of styrene in water (with Triton surfactant) emulsion solution at three power settings. The temperature decrease after 5-10 minutes was likely caused by the cooling effect of water/styrene vaporization. **(d)** Emulsion size at three different power levels using DLS and the respective physical images. For a similar solution composition, EVC yielded droplets around 100 nm (**Figure S8a**), while sonication produced ~140 nm droplets. For a similar solution composition, EVC yielded droplets around 100 nm (**Figure S8a**), while sonication produced ~140 nm droplets. The cloudiness of W/O emulsion can be attributed to higher droplet sizes (D_{avg} ~ 300 nm) compared to lower droplet sizes (~ 140 nm) for O/W emulsions. O/W emulsion initially showed larger droplet peaks and had turbid appearance which subsided over three weeks, and the solution became visibly clearer.

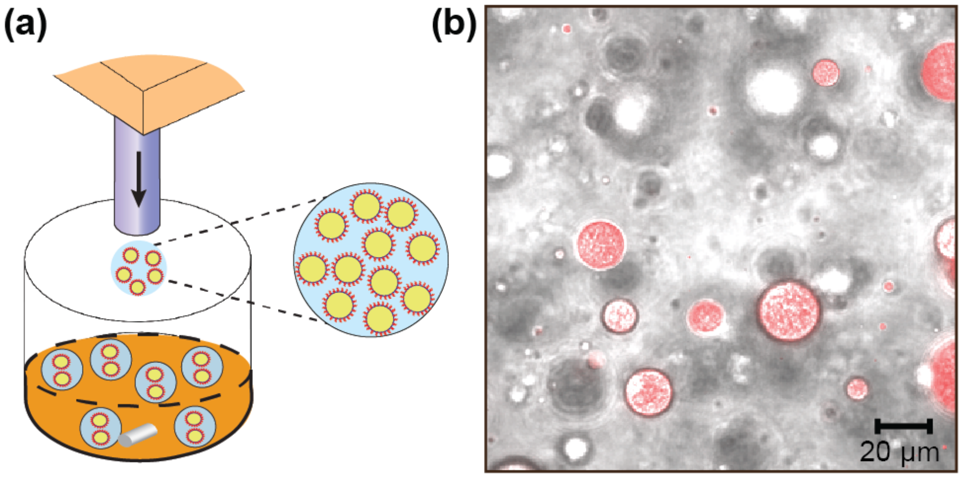

Figure S10| Complex emulsion synthesis. **(a) Schematic showing general principle of complex emulsion by dripping the water-in-oil emulsion on a second oil pool.**[11] To achieve this, the synthesized emulsion at the outlet is collected in another oil or oil-surfactant solution. We used styrene and krytox as the inner (Oil_1) and outer (Oil_2) oil phases, respectively. Styrene was condensed on a flowing aqueous mixture of Triton X-100 (75×cmc) and Rhodamine B dye. The resulting oil-in-water emulsion was then dripped onto a krytox bath and mixed with a stirring bar (400 rpm), forming a styrene-water-krytox emulsion. The high viscosity of krytox provided kinetic stability by delaying drop coalescence. **The resulting styrene-in-water-in-krytox complex emulsion was visualized using laser scanning confocal microscopy, and the images are shown in Figure S9b.** Conceivably, the channel configuration shown herein can be combined with microfluidics to create hybrid systems that can create novel types of particle systems. Such an aspect remains to be explored further. In all experiments the water surfactant solution was held at 10±0.5 °C and oil was heated to ~ 30 °C for vaporization. The collecting oil phase was held at room temperature.

Supplementary Movie Captions

Movie S1: Close-up view of the emulsified solution dripping from the channel and falling into the oil-surfactant solution for collection.

Movie S2: Condensation dynamics of five different oils on pure water bath and water-Triton X-100 solutions under stagnant flow conditions. The videos have been sped up by 300× their recording speed (30 fps).

Movie S3: Top view of the spreading dynamics of oil droplets (dyed red) gently deposited on the (8 ml) water-surfactant (Triton X-100) solution at room temperature (21°C). Each embedded video displays the speed at which it plays (e.g., 9×, 3×, 2×) compared to the recording speed (which was at 30 fps).

Reference:

[1] K. Giribabu, P. Ghosh, *Chem. Eng. Sci.* **2007**, *62*, 3057-3067.

[2] C. L. Yaws, *Yaws' Handbook of Properties for Aqueous Systems*, Knovel, **2012**.

[3] C. L. Yaws, *Yaws' critical property data for chemical engineers and chemists*, Knovel, **2012**.

[4] D. J. Kang, H. Bararnia, S. Anand, *ACS Applied Materials and Interfaces* **2018**, *10*, 21746-21754.

[5] Z. Wagner, M. Bendová, J. Rotrekl, S. Orvalho, *J. Solution Chem.* **2019**, *48*, 1147-1166.

[6] A. H. Demond, A. S. Lindner, *Environ. Sci. Technol.* **1993**, *27*, 2318-2331.

[7] aD. Kashchiev, *Nucleation*, Butterworth-Heinemann, **2000**; bS. Anand, K. Rykaczewski, S. B. Subramanyam, D. Beysens, K. K. Varanasi, *Soft Matter* **2015**, *11*, 69-80.

[8] D. Kashchiev, G. M. Van Rosmalen, *Cryst. Res. Technol.* **2003**, *38*, 555-574.

[9] D. Beysens, *The physics of dew, breath figures and dropwise condensation, Vol. 994*, Springer, **2022**.

[10] J. Christian, *The theory of transformations in metals and alloys*, Newnes, **2002**.

[11] H. Bararnia, University of Illinois at Chicago **2021**.
